# Epigenetic Therapies for Acute Myeloid Leukemia and Their Immune-Related Effects

**DOI:** 10.3389/fcell.2019.00207

**Published:** 2019-10-11

**Authors:** Valentina Gambacorta, Daniela Gnani, Luca Vago, Raffaella Di Micco

**Affiliations:** ^1^Unit of Senescence in Stem Cell Aging, Differentiation and Cancer, San Raffaele Telethon Institute for Gene Therapy (SR-TIGET), IRCCS San Raffaele Scientific Institute, Milan, Italy; ^2^Unit of Immunogenetics, Leukemia Genomics and Immunobiology, IRCCS San Raffaele Scientific Institute, Milan, Italy; ^3^Milano-Bicocca University, Milan, Italy; ^4^Unit of Hematology and Bone Marrow Transplantation, IRCCS San Raffaele Scientific Institute, Milan, Italy

**Keywords:** acute myeloid leukemia, epigenetics, therapy, immune activation, chromatin

## Abstract

Over the past decades, our molecular understanding of acute myeloid leukemia (AML) pathogenesis dramatically increased, thanks also to the advent of next-generation sequencing (NGS) technologies. Many of these findings, however, have not yet translated into new prognostic markers or rationales for treatments. We now know that AML is a highly heterogeneous disease characterized by a very low mutational burden. Interestingly, the few mutations identified mainly reside in epigenetic regulators, which shape and define leukemic cell identity. In the light of these discoveries and given the increasing number of drugs targeting epigenetic regulators in clinical development and testing, great interest is emerging for the use of small molecules targeting leukemia epigenome. Together with their effects on leukemia cell-intrinsic properties, such as proliferation and survival, epigenetic drugs may affect the way leukemic cells communicate with the surrounding components of the tumor and immune microenvironment. Here, we review current knowledge on alterations in the AML epigenetic landscape and discuss the promises of epigenetic therapies for AML treatment. Finally, we summarize emerging molecular studies elucidating how epigenetic rewiring in cancer cells may as well exert immune-modulatory functions, boost the immune system, and potentially contribute to better patient outcomes.

## Introduction

Acute myeloid leukemia (AML) is an aggressive blood cancer, characterized by the uncontrolled proliferation of poorly differentiated hematopoietic stem and progenitor cells. Even if disease prognosis has improved over the last decades, mainly thanks to decreased treatment-related mortality and wider use of allogeneic hematopoietic cell transplantation (allo-HCT) as consolidation therapy, prognostic classification of AML patients remains inaccurate and therapeutic options for high-risk patient are largely unsatisfactory (Dohner et al., 2015). Moreover, even if AML is often initially sensitive to chemotherapy, relapse events remain frequent either due to the emergence of chemotherapy-resistant clones (Lokody, 2014) or post-transplantation immune-escape mechanisms enacted by leukemic cells to evade donor immune system control (Vago et al., 2009; Toffalori et al., 2012; Christopher et al., 2018; Toffalori et al., 2019; Zeiser and Vago, 2019). Therefore, more effective combinatorial or alternative therapies are needed for AML patients.

In spite of being one of the tumors with the lowest mutational load, AML is a highly heterogeneous disease in terms of genetic background and clinical manifestation. Interestingly, although very few, mutations frequently hit genes encoding for proteins that regulate DNA methylation (such as DNMT3A, TET2, IDH1, and IDH2), affect post-translational modifications on histone tails (such as EZH2, ASXL1, and others), and drive three-dimensional chromatin conformation of cancer cells (such as CTCF and cohesin complex). In addition to mutations in epigenetic regulators, some of the fusion proteins that are known to drive abnormal transcriptional programs in AML act through aberrant expression or redirected specificity of epigenetic regulators. More broadly, global alterations in DNA methylation patterns and enhancer deregulation were recently linked to AML clonal expansion, further supporting the notion that epigenetic heterogeneity better explains leukemia identity compared to the genetic background (Corces et al., 2016; Li S. et al., 2016). The AML epigenome has therefore emerged as a new exciting target for drug discovery. Epigenetic modifications regulate chromatin states and gene expression changes without altering DNA sequences through distinct mechanisms including, among others, DNA methylation and post-translational modifications on histone tails. Our genome encodes for several enzymes that are able to deposit or remove chemical marks within specialized domains (writers and erasers, respectively) and to bind and recognize them (readers). The coordinated action of epigenetic writers, erasers, and readers are important for tight regulation of gene expression through downstream molecular effectors, thus contributing to both cancer development and progression. Unlike genetic alterations, epigenetic changes are dynamic and reversible, and the past decades have seen a dramatic increase of discoveries and clinical applications of small molecules targeting epigenetic modifiers with the final goal to restore normal epigenetic patterns in cancer cells. Compared to other targeted approaches, epigenetic therapies may potentially reduce the emergence of molecular resistance and clinical recurrence by simultaneously impacting on distinct cellular pathways. In addition, besides the aforementioned cell autonomous mechanisms of action, epigenetic therapies may have immunomodulatory properties and further enhance the sensitivity of cancer cells to immune therapies. In this Review, we provide an overview of epigenomic changes involved in AML pathogenesis and describe the mechanisms of action of epigenetic drugs currently in use or under investigation for AML treatment. Table 1 summarizes the status of epigenetic therapies for AML from preclinical testing to clinical approval. Finally, we discuss emerging concepts and promising new therapeutic approaches based on the interplay between epigenetic therapies and immune system modulation.

**TABLE 1:** Epigenetic therapies in AML.

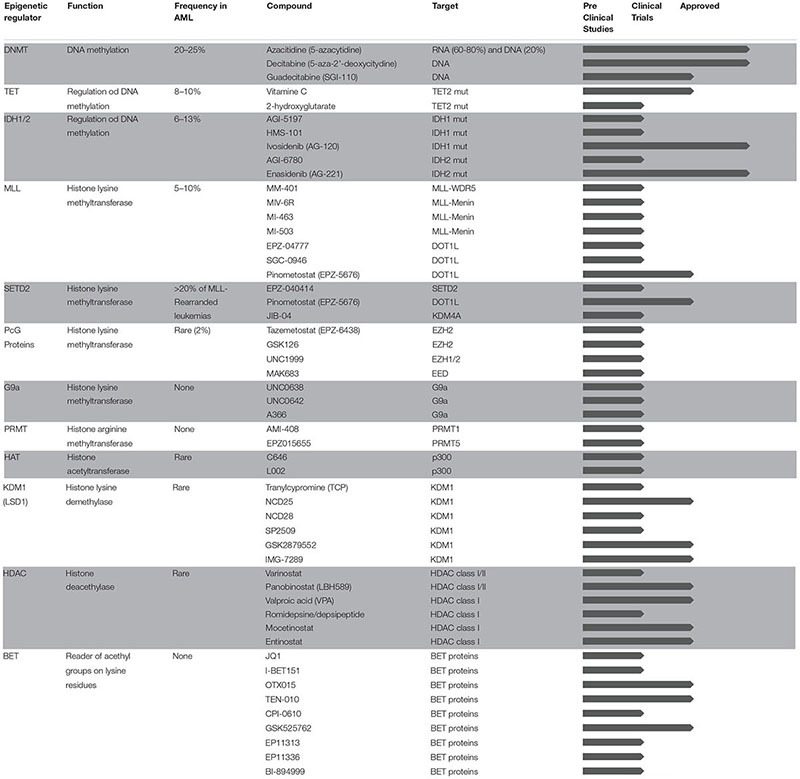

## Epigenome Deregulation and Epigenetic Therapies for AML Treatment

### Epigenome Writers

The enzymes able to catalyze modifications on DNA or histone proteins are named “epigenetic writers” and can be classified into enzymes impacting on DNA methylation, histone methyltransferases (HMTs), and histone acetyltransferases (HATs). The first category includes all the proteins that directly contribute to the regulation of DNA methylation (DNA methyltransferases; DNMTs). The HMT category comprises lysine methyltransferases (KMTs) and arginine methyltransferases (PRMTs). Methylation of histone tails promotes both gene activation and repression depending on the modified histone residue. In detail, methylation of H3K4, H3K36, H3K79, and H4R3 is generally associated to transcriptional activation, while methylation of H3K9, H3K27, and H4K20 induces transcriptional repression. Finally, HATs are epigenetic writers responsible for acetylation of lysine residues on nucleosomes, which is associated to open chromatin and activation of gene expression.

#### DNA Methylation

##### DNMTs

DNA methylation is a heritable epigenetic mark mainly contributing to gene repression. Specifically, DNA methylation levels are under the control of enzymes able to modulate the addition (by DNA methyl transferases; DNMTs) or the removal (by the indirect action of TET and IDH1/2 proteins) of methyl groups to cytosine or adenine residues. Besides the essential physiological functions, alterations in DNA methylation pattern have been extensively described in different cancer types including AML. Importantly, it has been reported that genetic alleles and epialleles (as assessed by genome-wide DNA methylation profile) can evolve independently from each other during AML progression and that the DNA methylation patterns can be used to stratify patients and predict clinical outcome (Li S. et al., 2016; Figueroa et al., 2010b). Interestingly, these changes are mostly determined by DNA methylation of non-promoter regulatory regions rather than at the level of gene promoters. Indeed, different methylation patterns of enhancer elements can also precisely distinguish normal blood cell from leukemic cells in different disease stages (Figueroa et al., 2010b; Qu et al., 2017). Moreover, in AML cells, aberrantly methylated sites, mainly residing in gene bodies and enhancer elements, often display features associated with aged blood cells including, among the others, chromatin changes in immune and cell-adhesion genes (Glass et al., 2017; Adelman et al., 2019). One possible driver of the epigenetic reprograming of AML blasts can be the presence of somatic mutations affecting DNMTs. DNMT3A, a *de novo* methyltransferase, is mutated in 20–25% of AML patients (Thol et al., 2011a; Cancer Genome Atlas Research et al., 2013; Papaemmanuil et al., 2016) and linked by several studies to decreased overall survival (Thol et al., 2011a; Ribeiro et al., 2012). Notably, the majority of somatic DNMT3A mutations occurs at arginine (R) 882 and lead to decreased catalytic activity and DNA binding affinity. However, the molecular mechanisms by which DNMT3A mutations favor leukemia occurrence are still unclear. It has been originally reported that mutant DNMT3A alters the expression of genes involved in key cellular pathways including apoptosis and hematopoietic stem cell (HSC) self-renewal (Tadokoro et al., 2007; Thol et al., 2011a, b). Deletion of DNMT3A in mice was shown to impair HSC differentiation and to increase the number of phenotypically defined HSCs although no signs of overt malignancy were observed upon transplantation of DNMT3A-deleted HSCs, suggesting that additional alterations may be required for leukemia development (Challen et al., 2011).

Given the pivotal role of DNA methylation in remodeling AML epigenome at both promoters and distal regulatory elements, DNMTs emerged as attractive therapeutic targets to restore normal DNA methylation patterns in leukemic blasts. Two nucleosidic epigenetic compounds inhibiting DNMT activity, azacytidine (5-azacytidine) and decitabine (5-aza-2′-deoxycytidine) (DNMTi), are currently in clinical use for myeloid malignancies. Azacytidine, upon conversion to decitabine, incorporates into newly synthetized DNA, thwarting the binding of DNMTs. Of notice, azacytidine is predominantly incorporated into RNA with a more evident effect on gene translation (Navada et al., 2014). Chemical DNMT inhibition significantly alters DNA methylation patterns with consequent induction of cell cycle arrest, DNA damage accumulation, apoptosis, differentiation, and immune cell activation (Wouters and Delwel, 2016). Both azacytidine and decitabine initially entered standard clinical practice for the treatment of myelodysplastic syndrome (MDS) and AML patients with low blast count. In a subsequent phase II clinical trial, decitabine showed acceptable tolerability and efficacy also in AML patients older than 60 with >30% of blasts and not eligible for intensive chemotherapy (Cashen et al., 2010). Moreover, a phase III trial in older or unfit AML patients reported higher response rate and survival advantage in patients treated with decitabine compared with current standard of care (low-dose cytarabine or supportive care) (Kantarjian et al., 2012). More recently, next-generation DNMT inhibitors with improved stability such as guadecitabine (SGI-110) have been developed and tested in clinical trials with promising results (Issa et al., 2015; Stein and Tallman, 2016; Garcia-Manero et al., 2019). However, to date, the efficacy of DNMTi as single agents for AML treatment is limited, possibly due to the fact that targeting a single layer of epigenetic deregulation (e.g., DNA methylation) cannot be sufficient to reach a complete rescue of the epigenetic landscape of leukemic blasts. On this purpose, several studies reported promising preliminary results from combinatorial treatments of DNMTi with other epigenetic drugs including HDAC inhibitors (HDACi; discussed below), or with agents commonly in use for AML patients such as FLT3 inhibitors, lenalidomide, and antibody–drug conjugates (Gardin and Dombret, 2017). To date, the most promising combination for AML treatment is the one with azacytidine or decitabine and venetoclax (ABT-199), an inhibitor of the anti-apoptotic protein BCL-2. Mechanistically, venetoclax in combination with hypomethylating agents leads to a metabolic rewiring that suppresses oxidative phosphorylation and selectively triggers apoptosis in leukemic stem cells (Pollyea et al., 2018). From a clinical standpoint, the combinatorial treatment of venetoclax plus DNMTi was effective and well tolerated in elderly AML patients not eligible for intensive chemotherapy (DiNardo et al., 2019).

##### TET

Another layer of epigenetic regulation of DNA is the oxidation of 5mC (5hmC), which indirectly prevents the addition of methyl groups on cytosine by DNMTs. This modification is catalyzed by the Ten-Eleven-Translocation (TET) enzymes and depends on the action of isocitrate dehydrogenase 1/2 (IDH1/2) proteins, which in turn produce α-ketoglutarate (α-KG) to stimulate TET activity. Somatic mutations in both these classes of enzymes cause aberrant DNA hypermethylation mainly occurring at gene promoters. Specifically, TET2 mutations affect 8–10% of patients with *de novo* AML (Thol et al., 2011a; Cancer Genome Atlas Research et al., 2013; Papaemmanuil et al., 2016) and are associated to a global reduction of 5hmC. This deregulation leads to alterations in several biological processes including differentiation and proliferation (Figueroa et al., 2010a; Moran-Crusio et al., 2011; Quivoron et al., 2011). As previously reported for DNMTs, TET2 activity is not only limited to promoter regions. In fact, genome-wide mapping of TET2 binding sites revealed the presence of this protein also in regulatory regions such as enhancers where it is fundamental for the recruitment of hematopoietic transcription factors including IRF, CEBP, GATA, and HOX proteins (Rasmussen et al., 2015). Also, TET2 loss-of-function mutations have been very recently reported to be important for immune cell activation and for leukemic blast differentiation. Thus, drugs acting on TET2 function (including vitamin C and 2-hydroxyglutarate) can both halt leukemia progression and induce antitumor immune cell-mediated response (Tyrakis et al., 2016; Cimmino et al., 2017; Fraietta et al., 2018).

##### IDH1/2

Present in around 6 to 13% of AML patients (Ward et al., 2010), IDH1/2 mutations lead to the production of 2-hydroxyglutarate (2-HG), are mutually exclusive with TET2 alterations and trigger a hypermethylation signature almost completely overlapping to the one characteristic of TET2 mutant cells. Given the relatively high frequency of IDH mutations in AML, over recent years, several small molecules targeting mutated IDH1/2 have been developed, and, upon successful testing in clinical trials, some of them have already been approved by the Food and Drug Administration.

AGI-5198 was the first potent inhibitor of mutant IDH1, initially described in the context of glioma cells. Blockade of IDH1/IDH2 mutant forms reduces 2-HG production and H3K9me3 levels, thus inducing cancer cell differentiation (Rohle et al., 2013). Another IDH1 inhibitor, ivosidenib (AG-120), showed similar ability to promote differentiation of mutated leukemia cells and received FDA approval last year thanks to the very high response rate in relapse/refractory AML patients (DiNardo et al., 2018). Furthermore, a computational drug screening identified another selective inhibitor of mutant IDH1 (HMS-101), which was also effective in inhibiting leukemia growth *in vitro* (Chaturvedi et al., 2013).

Regarding inhibitors of IDH2, AGI-6780 and enasidenib (AG-221) showed high selectivity for the mutant form of IDH2 sparing the wild-type isoform and have been shown to induce differentiation in AML cells both *in vitro* and in xenograft models of AML (Wang et al., 2013; Yen et al., 2017). The efficacy of enasidenib was also confirmed in relapse/refractory AML patients carrying IDH2 mutation, leading to its recent clinical approval (Stein et al., 2017, 2019).

### Histone Methylation

#### MLL

Part of the Drosophila Trithorax family of proteins, the lysine methyltransferase MLL (or KMT2A) is involved in the methylation of H3K4 residue (H3K4me1/2/3), a transcriptional activation mark. MLL translocations with multiple factors as well as MLL partial tandem duplications (MLL-PTD) are observed with frequencies ranging from 5 to 10% in AML patients, and are associated with poor prognosis (Schichman et al., 1994; Caligiuri et al., 1998; Dohner et al., 2002; Milne et al., 2002). To date, more than 80 different MLL fusion partners have been identified. MLL translocations result in fusion oncoproteins, which lack the C-terminal SET domain of MLL (responsible for the H3K4 methylation activity) and gain domains from the different fusion partners. Several small molecules inhibiting MLL complexes and cofactors have been developed, and some of them are currently in clinical trials for the treatment of MLL-rearranged leukemias. MM-401 blocks the MLL-WDR5 fusion gene, leading to proliferation arrest and myeloid differentiation of leukemia cells while sparing normal hematopoietic stem/progenitor cells (Cao et al., 2014). Through high-throughput screening and structure-based chemical development, other small molecules targeting the MLL-cofactor interactions were discovered and optimized to target MLL-Menin oncoprotein. Specifically, MIV-6R, MI-463 and MI-503 efficiently and selectively suppressed MLL-rearranged leukemia growth both *in vitro* and *in vivo* (Grembecka et al., 2012; He et al., 2014; Borkin et al., 2015). In the last years, some studies also revealed that in order to drive tumorigenesis, MLL fusion proteins require DOT1L, a unique HMT that specifically catalyzes H3K79 methylation (Okada et al., 2005; Bernt et al., 2011; Jo et al., 2011; Nguyen et al., 2011). Upon DOT1L blockade, levels of H3K79 methylation drop, and this in turn blocks the expression of MLL fusion proteins target genes including HOXA9 and MEIS2 and trigger selective death in MLL-rearranged cell lines harboring DOT1L-recruiting fusion partners (Daigle et al., 2011, 2013; Chen et al., 2013). Based on these findings, the DOTL1 inhibitor EPZ-5676 is currently being tested in several early phase clinical trials for MLL-rearranged leukemias. Published results from a phase I trial revealed an acceptable safety profile and an interesting overall response rate in patients with advanced hematological cancers with MLL rearrangements (Stein et al., 2018). Moreover, the potential therapeutic effect of DOT1L inhibitors has been described in other genetically defined AML subgroups such as the ones bearing partial tandem duplications within the MLL gene (MLL-PTD). Indeed, the MLL-PTD leukemias share critical biological features with MLL-rearranged leukemia, including the requirement of DOT1L for their oncogenic activity and high expression levels of HOXA-cluster genes. Given these similarities, MLL-PTD positive leukemias showed high sensitivity to EPZ-5676 in both *in vitro* and *in vivo* models (Kuhn et al., 2015) and were therefore also included in the phase I clinical trial mentioned above (Stein et al., 2018).

#### SETD2

SETD2 is a lysine methyltransferase responsible for tri-methylation of the transcriptional elongation mark H3K36 that has been found to be mutated in different tumor types, including hematological malignancies (Mar et al., 2014; Zhu et al., 2014; Moffitt et al., 2017; Lin et al., 2018). Mutations in SETD2 result in global reduction of H3K36me3 in tumor cells, which in turn exhibit impaired DNA damage signaling and fail to activate the tumor suppressor p53 (Carvalho et al., 2014). Interestingly, SETD2 loss-of-function mutations have been identified in more than 20% of leukemia patients with MLL gene rearrangement (Bu et al., 2018), contributing to both initiation and leukemia progression by enhancing the self-renewal potential of leukemic stem cells. According to some studies, SETD2 appears to be required for sustaining the functionality and the correct differentiation of HSCs (Zhang et al., 2018; Zhou et al., 2018). Initially considered as a tumor suppressor, recent data suggest that SETD2 may as well act as an oncogene. In fact, if partial SETD2 loss accelerates leukemogenesis and induces drug resistance, complete SETD2 loss delays leukemia progression. This opposite behavior of SETD2 may rely on independent H3K36me3 functions that need to be further investigated (Skucha et al., 2019). Interestingly, the DOT1L inhibitor EPZ-5676 induced differentiation and cell death in a MLL-rearranged leukemia model bearing SETD2 mutation (Bu et al., 2018). Interestingly, a recent study showed that the treatment with JIB-04, an inhibitor of the lysine demethylase KDM4A, was able to restore H3K36 methylation levels and chemotherapy sensitivity to SETD2-mutant leukemias (Mar et al., 2017).

#### Polycomb Proteins

First discovered in Drosophila, Polycomb group (PcG) proteins are constituents of two chromatin-remodeling complexes (PRC1 and PRC2) that act as transcriptional repressors, regulating key biological processes including cell proliferation, differentiation, and stem cell plasticity (Margueron and Reinberg, 2011; Morey et al., 2015). PRC1 composition is variable, with two core components, RING1A and RING1B (Margueron and Reinberg, 2011), and several accessory components. The PRC1 complex acts through mono-ubiquitination of histone H2A lysine 119. One of the accessory PRC1 components, BMI1, has been shown to have a role in controlling the self-renewal of normal and leukemic stem cells in AML (Lessard and Sauvageau, 2003). Based on the role of BMI1 in HSC homeostasis, overexpression of this protein is frequently found in patients with hematologic disorders (Mihara et al., 2006; Chowdhury et al., 2007; Rizo et al., 2009).

PRC2 is a multi-subunit complex consisting in four core constituents: drosophila enhancer of zeste homolog 2 (EZH2), embryonic ectoderm development (EED), suppressor of zeste 12 homolog (SUZ12), and human retinoblastoma binding protein 4 (RBBP4). Furthermore, accessory molecules favoring PRC2 recruitment and stabilization include JARID2 and YY1. EZH2 is the catalytic subunit of the PRC2 complex and is involved in mono, di, and tri-methylation of H3 lysine 27 (H3K27me1/2/3), leading to chromatin compaction and transcriptional repression of target genes. Acting both as oncogene and tumor suppressor, EZH2 plays contrasting roles in AML pathogenesis (Basheer et al., 2019). Frequently overexpressed in solid tumors, lymphomas (Morin et al., 2010) and in myeloid malignancies, EZH2 has a dual role: in fact, it can potentially act as a tumor suppressor, as suggested by a number of identified loss-of-function mutations (Nikoloski et al., 2010), or promote tumorigenesis and be linked to inferior patient outcome when overexpressed (Grubach et al., 2008; Nikoloski et al., 2010; Xu et al., 2011). Consistent with the oncogenic role of EZH2, inhibition of EZH2 in a mouse model of leukemia resulted in reduced number of leukemic stem cells and impaired leukemia growth (Fujita et al., 2018). Accordingly, the differentiation and growth inhibitory effects of the EZH2 silencing on AML cells further corroborate the oncogenic function of EZH2 in AML (Tanaka et al., 2012). Besides EZH2, deregulation of other members of PRC2 components including ASXL1, JARID2, EED, and SUZ12 has been implicated in the development and propagation of hematological malignancies, including AML (Boultwood et al., 2010; Chou et al., 2010; Metzeler et al., 2011; Beekman et al., 2012; Puda et al., 2012; Schnittger et al., 2013; Micol et al., 2014; Paschka et al., 2015; Qi et al., 2017).

Given this evidence, several small molecules inhibiting PRC2 complex and its components have been developed and tested in preclinical and clinical settings. Both GSK126 and EPZ-6438 (tazemetostat) are currently in early stage clinical trials for a variety of hematological malignancies including lymphoma. Recent studies show that PRC2 acts in parallel with MLL rearrangements to sustain leukemia growth (Neff et al., 2012; Shi et al., 2013). Based on this, UNC1999, an EZH1/EZH2 dual inhibitor, showed its efficacy in suppressing AML growth in both *in vitro* and *in vivo* experiments in MLL-rearranged AML models reverting the repressed PRC2 target gene signatures to an active status (Xu et al., 2015). In addition to inhibitors of the PRC2 enzymatic domain, small molecules targeting other components of the complex have been developed. By directly binding H3K27, EED is essential for the HMT activity of PRC2. The small molecule MAK683 disrupts EED-EZH2 protein–protein interaction, preventing H3K27 trimethylation. This has been associated with decreased tumor cell proliferation in diffuse large B-cell lymphoma (DLBCL) cell lines and to tumor regression in a mouse lymphoma xenograft model (Qi et al., 2017). While a clinical trial is testing MAK683 in lymphoma patients (NCT02900651), the clinical potential of PRC inhibitors in AML patients remains to be determined. Recently, the emergence of resistance to EZH2 inhibition has been reported in preclinical models of lymphoma. Interestingly, the majority of the pathways involved in the establishment of resistance mechanisms to epigenetic therapies are in common between different drugs and tumor types. This is the case of inhibitors of EZH2 and BET proteins (discussed below). Indeed, in response to each treatment, inhibitor-resistant cells showed the constitutive activation of the phosphoinositide-3-kinase (PI3K) pathway (Kurimchak et al., 2016; Bisserier and Wajapeyee, 2018). Other works described that the acquisition of genetic mutations in EZH2 gene confers resistance to EZH2 inhibition in the same diffuse large B-cell lymphoma cell lines (Baker et al., 2015; Gibaja et al., 2016).

#### G9a

G9a is a lysine methyltransferase belonging to the Su(var)3-9 family, which catalyzes the reaction of mono/di-methylation at H3K9 (H3K9me1/2) triggering gene repression (Shankar et al., 2013). Even if there are no mutations in AML targeting the G9a gene, recent studies reported that loss of this protein suppressed leukemogenesis in a mouse model of leukemia induced by HOXA9, an oncoprotein overexpressed in 50–70% of AML patients and for which there are no currently available inhibitors (Lehnertz et al., 2014). Two G9a inhibitors, UNC0638 and UNC0642, the latter displaying an improved pharmacokinetics, showed remarkable cytotoxicity against AML cell lines (Vedadi et al., 2011; Liu et al., 2013). A-366, a recently developed peptide-competitive inhibitor of G9a, displayed a reduced toxicity profile compared to UNC0638 and UNC0642 in human prostate cancer cell lines (Sweis et al., 2014). The role of G9a inhibitors in AML patients remains to be tested.

#### PRMT

Arginine residues within histone tails (H3 and H4) can be regulated through methylation by protein arginine methyltransferases (PRMTs). Protein arginine methylation is an abundant post-translational modification, which regulates a plethora of pathways including signal transduction, gene transcription, DNA repair, and mRNA splicing. Crosstalk occurring between PRMT and KMT is necessary to establish appropriate patterns of histone methylation (Hyllus et al., 2007; Iberg et al., 2008). Among PRMT targets, there are also non-histone substrates, including AML1 and ASH2L, suggesting that alterations in the activity of PRMTs may have widespread effects. Even if no mutations have been found in PRMT genes, overexpression of these proteins is often present in various cancer types including leukemia rendering these enzymes particularly intriguing as therapeutic targets. Within the PRMT family, PRMT1 catalyzes asymmetric di-methylation of arginine 3 of H4 (H4R3me2) that results in transcriptional activation, by promoting p300-mediated acetylation of K8 and K12 on H4 (Wang et al., 2001; An et al., 2004). In normal hematopoiesis, PRMT1-mediated methylation of the transcription factor AML1 enhances its ability to activate the transcription of several target genes during hematopoietic differentiation (Zhao et al., 2008). Moreover, PRMT1 has also been found to methylate ASH2L, which has been suggested to act as an oncoprotein (Butler et al., 2011).

The interest in targeting PRMTs for AML therapy comes from several studies reporting specific requirement of PRMT1 for leukemogenesis in some genetic subsets of AML, including those with AML1-ETO and MLL rearrangements. In particular, PRMT1 has been found to be required for leukemia initiation by the fusion proteins MLL–GAS7 and MOZ–TIF2 and its silencing was able to block leukemia transformation (Cheung et al., 2016). Moreover, PRMT1 methylates the AE9a isoform of the AML1-ETO leukemogenic fusion protein and activates AE9a target genes, enhancing proliferation of hematopoietic progenitor cells. Accordingly, knockdown of PRMT1 suppresses the self-renewal capability of AE9a-positive cells, suggesting a role of PRMT1 in regulating leukemogenesis (Shia et al., 2012). Along these lines, preclinical studies reported that small molecules inhibiting PRMT1 (AMI-408) suppressed the growth of both human AML cell lines and leukemia mouse models (Dillon et al., 2012; Cheung et al., 2016). Similar cytotoxic effects were reported in lymphoma models overexpressing PRMT5 upon treatment with the PRMT5 specific inhibitor EPZ015666 both *in vitro* and *in vivo* (Chan-Penebre et al., 2015). ERZ015666 was also recently reported to rescue the differentiation block of human leukemic cells *in vitro* and in a mouse model of MLL-rearranged leukemia (Kaushik et al., 2018).

## Histone Acetylation

Although poorly described so far, alterations of enzymes belonging to HAT family have been detected in AML. In particular, several translocations involving HAT proteins as fusion partners have been described, including t(8;16) (p11;p13) and t(8;22) (p11;p13). These rearrangements generate the MOZ-CBP and the MOZ-P300 fusion proteins respectively and both have been shown to contribute to leukemogenesis (Borrow et al., 1996; Kitabayashi et al., 2001). Inhibitors targeting components of this family of enzymes have been developed and tested in preclinical models. In particular, the P300 inhibitors C646 and L002 showed great selectivity in inducing proliferation arrest of leukemia and lymphoma cell lines (Gao et al., 2013; Yang et al., 2013).

### Epigenetic Erasers

Enzymes able to remove epigenetic marks are identified as “epigenetic erasers.” There are two main groups of proteins belonging to this class: histone demethylases (HDMs) and histone deacetylases (HDACs).

#### HDM

The first group of proteins can be, in turn, classified into two other big families: amino oxidase homolog lysine demethylase 1 (KDM1) and JMJC domain containing HDMs. Mutations of genes belonging to the latter family are rare in AML and mainly limited to the ones occurring in lysine demethylase 6A (KDMT6A) (Cancer Genome Atlas Research et al., 2013). The lysine demethylase KDM1 (also known as LSD1) demethylates di- and mono-methylated K4 on histone H3, reducing the levels of H3K4me3, normally associated with active gene transcription. LSD1 has been shown to affect a wide range of transcriptional programs, acting either as a transcriptional repressor or as an activator depending on the cellular context (Maiques-Diaz and Somervaille, 2016). Pharmacological inhibition or genetic depletion of LSD1 induces differentiation of MLL-driven AML stem cells and of other genetically defined AML subtypes (Maiques-Diaz et al., 2018). A recent study showed that the LSD1 inhibitor tranylcypromine (TCP) induced the expression of myeloid differentiation genes in AML cells and that combination of TCP with ATRA exerted a potent anti-leukemic effect (Schenk et al., 2012). Recently, two novel LSD1 inhibitors, NCD25 and NCD38, were identified for their ability to halt leukemia growth and induce myeloid differentiation. In particular, NCD38 was shown to reactivate clusters of enhancer elements (e.g., super-enhancers) that control hematopoietic genes and that are abnormally silenced by LSD1 during leukemia progression (Sugino et al., 2017). If the treatment with the LSD1 inhibitor SP2509 demonstrated high efficacy in blocking leukemia growth, the co-treatment with a specific histone deacetylase (HDAC) inhibitor, panobinostat, was synergistically lethal against both primary and leukemia cell lines (Fiskus et al., 2014). The LSD1 inhibitors GSK2879552 and IMG-7289, alone or in combination with all-trans retinoic acid therapy (ATRA), showed promising activity against AML *in vitro* (Smitheman et al., 2019), leading to two ongoing phase I trials for patients with relapsed/refractory AML (NCT0217782, NCT02842827) and to a phase I/II trial for MDS patients (NCT02929498).

#### HDAC

Given the importance of histone acetylation in regulating the expression of many genes, it is not surprising that enzymes able to regulate this modification are frequently hit by alterations in different tumor types including hematological malignancies. Thus, HDAC alterations can be linked to both silencing of tumor suppressor genes and activation of oncogenic processes altering the cell cycle progression, activation of the DNA damage response (DDR) pathway, apoptosis, and many others. Although HDAC somatic mutations have been identified so far in several solid tumors with a relatively high frequency (Stark and Hayward, 2007; Taylor et al., 2011), they are rare in AML patients. However, the contribution of HDAC to AML pathogenesis has been linked to aberrant recruitment of these enzymes by myeloid oncoproteins such as AML1-ETO, PML-RARA, and EVI1 (Izutsu et al., 2001; Senyuk et al., 2002; Hug and Lazar, 2004; Falkenberg and Johnstone, 2014).

In the last years, several clinical trials have been conducted with HDACi in patients with MDS and AML. The HDACi vorinostat and panobinostat are among the earliest approved by FDA for treatment of cutaneous T cell lymphoma and multiple myeloma, given their capacity to induce cancer cell differentiation. Second-generation HDACi are currently in use in several clinical trials for different tumors, including relapsed AML (Finazzi et al., 2013; Li Y. et al., 2016). In the past years, several studies addressed the molecular effects of HDAC inhibition in cancer cells. Microarray analyses revealed transcriptional changes of a large number of genes (approximately 10–20% of the genome), including proapoptotic inducers and genes involved in cell cycle arrest in leukemic cell lines after exposure to different HDACi (Peart et al., 2005). According to the literature, HDACi exert their anti-proliferative effects via induction of apoptosis, regulation of different signaling pathways (Bali et al., 2005; Insinga et al., 2005; Nebbioso et al., 2005; Xu et al., 2006), and activation of the DDR pathway in oncogene-expressing cells (Di Micco et al., 2011). Of note, it has also been reported that HDAC inhibition in normal and leukemic cells induces DDR activation in the absence of physical DNA lesions. Specifically, chromatin remodeling induced by these inhibitors may directly activate DDR through the increased phosphorylation of the histone H2AX (γH2AX) and/or through ROS production (Gaymes et al., 2006; Petruccelli et al., 2011).

Valproic acid (VPA) is a short-chain fatty acid acting as a powerful HDACi that causes hyperacetylation of the N-terminal tails of H3 and H4 histones by inhibiting the catalytic activity of class I HDACs (Gottlicher et al., 2001). VPA has a wide range of effects on leukemic cells. By analyzing the effects of VPA treatment on AML patient blasts (Rucker et al., 2016) identified a signature enriched for pathways implicated in cell cycle arrest, apoptosis, and DNA repair. However, indirect effects of VPA on the reactivation of antitumor immune response may also be considered. In addition to VPA, other HDACi including romidepsin/depsipeptide, mocetinostat, and entinostat, have been tested in phase 1/2 studies for leukemia treatment. However, HDACi have shown better results when used in combination with other agents with known anti-leukemia activity (Garcia-Manero et al., 2012; Gojo et al., 2013; Kirschbaum et al., 2014). Panobinostat, already approved for the treatment of multiple myeloma, is now under clinical investigation for AML patients. As a single agent, panobinostat showed modest anti-leukemic activity in clinical trials for myeloid malignancies (Giles et al., 2006; DeAngelo et al., 2013; Schlenk et al., 2018). On the other hand, *in vitro* studies showed that combinations of panobinostat with other treatments or epigenetic drugs could have synergistically lethal effects on AML cells (Fiskus et al., 2014). However, results from a recent clinical trial of panobinostat in combination with intensive chemotherapy did not show any clinical improvement and was accompanied by increased toxicities in treated AML patients (Schlenk et al., 2018). Overall, as HDACs also deacetylate numerous non-histone proteins, the widespread effects on the whole cellular proteome should be better investigated and taken into consideration to fine-tune successful therapies.

### Epigenetic Readers

The group of proteins able to recognize and bind post-translational modifications are called “epigenetic readers.” These proteins have specialized domains able to recognize a variety of nucleosome modifications acting directly on the transcription or indirectly by serving as scaffold for the recruitment of other epigenetic regulators. The cooperation between these proteins and the chromatin-modifying enzymes is therefore fundamental for gene expression patterns and deregulation of chromatin readers has been frequently reported in cancer.

#### BET

Bromodomain and extra-terminal domain (BET) proteins are a class of chromatin readers that act by binding histone and non-histone acetyl groups at lysine residues (Wu and Chiang, 2007). Among these, BRD4 has emerged as a key regulator of transcriptional networks in development and cellular differentiation (Di Micco et al., 2014; Dey et al., 2019) as well as a key player in driving aberrant transcriptional programs in cancer cells (Asangani et al., 2014; Filippakopoulos and Knapp, 2014; Shu et al., 2016; Fontanals-Cirera et al., 2017). In the context of AML, BRD4 sustains the expression of c-MYC to promote aberrant self-renewal (Zuber et al., 2011). More recently, it has been reported that BRD4 binds and recognizes specialized regions of H3K27 acetylation called “super-enhancers,” which control several lineage-specific genes and can be hijacked by tumor cells to express critical oncogenes (Loven et al., 2013). In addition to its originally described chromatin reader activity, BRD4 was recently shown to have HAT activity that results in chromatin relaxation and is conserved across species (Devaiah et al., 2016). Based on these findings, and on the detrimental effects of BRD4 depletion on AML proliferation, many inhibitors targeting BET proteins have been designed and tested against several tumor types, including leukemia (Perez-Salvia and Esteller, 2017). Among these, the small molecules JQ1 and the I-BET151 have been shown to be highly effective in inducing cell cycle arrest and apoptosis of MLL-rearranged leukemia cells both *in vitro* and *in vivo*. These inhibitors act by displacing BRD4 from regulatory elements and blocking the RNA Pol II mediated transcriptional elongation at the level of specific oncogenes including c-MYC, BCL2, and CDK6 (Dawson et al., 2011; Zuber et al., 2011). Similarly, the BET inhibitor OTX015 showed the ability to induce apoptosis in a variety of leukemic cell types (Coude et al., 2015). Several clinical trials testing BET inhibitors are currently ongoing; the small molecule RO6870810/TEN-010 (a more stable derivate of JQ1) has been tested in a recently completed phase I trial for the treatment of refractory AML and MDS (NCT02308761), and a phase II trial testing the BET inhibitor CPI-0610 in combination with ruxolitinib is open for patients with myelofibrosis (NCT02158858). On the same line, the BRD4 inhibitor GSK525762 entered early phase clinical trials for patients with relapsed refractory hematological malignancies. Importantly, a recent work by Gerlach et al. (2018) identified a novel BET inhibitor (BI-894999) belonging to the family of [1,2,4]triazolo[4,3-a]pyrazines. Distinct in structure compared to other BET inhibitors, BI-894999, although regulating the same genes as JQ1, showed a higher efficacy in killing AML cells derived from primary samples and xenograft models. In addition, the combination with an inhibitor of CDK9, a component of the transcriptional elongation complex, strongly enhanced its antitumor effects (Gerlach et al., 2018). Even if the interest in targeting BET proteins for cancer treatment keeps growing, there is still a lack of valuable BET transgenic animal models to elucidate the toxic effects and the mechanisms of action of BET inhibitors. Of note, an inducible BRD4 RNA interference animal model showed that BRD4 depletion causes toxicity in several organs and induces intestinal stem cell depletion (Bolden et al., 2014). Furthermore, even though still largely unexplored, one of the best-characterized mechanisms of tumor resistance to epigenetic therapy includes resistance to BET inhibitors. In particular, it has been reported in a MLL-AF9;Nras(G12D) AML mouse model that resistance to BET inhibition involves chromatin remodeling that in turn activates the WNT signaling pathway (Rathert et al., 2015). In addition, elevated levels of ERK/PI3K activity were shown to mediate BET inhibitor resistance (Kurimchak et al., 2016) providing a rationale for combinatorial strategies that simultaneously target BET proteins and receptor tyrosine kinases (RTKs) (Wyce et al., 2018) or the PI3K pathway (Stratikopoulos et al., 2015).

## Enhancer Deregulation in AML

The systematic characterization of regulatory regions in normal and AML blasts led to the identification of clusters of transcriptionally active chromatin domains with putative or proven enhancer activity for expression of leukemic genes (defined as “active enhancers” or “super-enhancers”). Enhancer elements present in normal blood cells may accumulate mutations that generate new binding sites for transcription factors and establish new enhancers driving leukemia oncogene expression (Mansour et al., 2014). Similarly, pre-existing enhancers may be inverted, translocated, or even undergo duplications to drive the aberrant upregulation of oncogenes or to suppress the expression of tumor suppressor genes. In addition, mutations in CTCF, a key player in driving the 3D chromatin organization (Zuin et al., 2014), may favor novel promoter/enhancer interactions to sustain leukemic cell proliferation. Recently, it was reported that a blood enhancer cluster (BENC) known to regulate the expression of c-MYC in normal HSCs can be hijacked by leukemic stem cells in mouse models of leukemia and displays an accessible chromatin profile even in primary AML samples (Bahr et al., 2018). Furthermore, charting the enhancer landscape by chromatin accessibility in single AML cells revealed a peculiar “regulome” profile that paralleled the developmental stage of disease, with the acquisition of a closed chromatin conformation at the level of HOXA genes, which have been suggested to be important in the first step of leukemogenesis (Corces et al., 2016).

## Epigenetic Therapies and Immune Response

**FIGURE 1:**
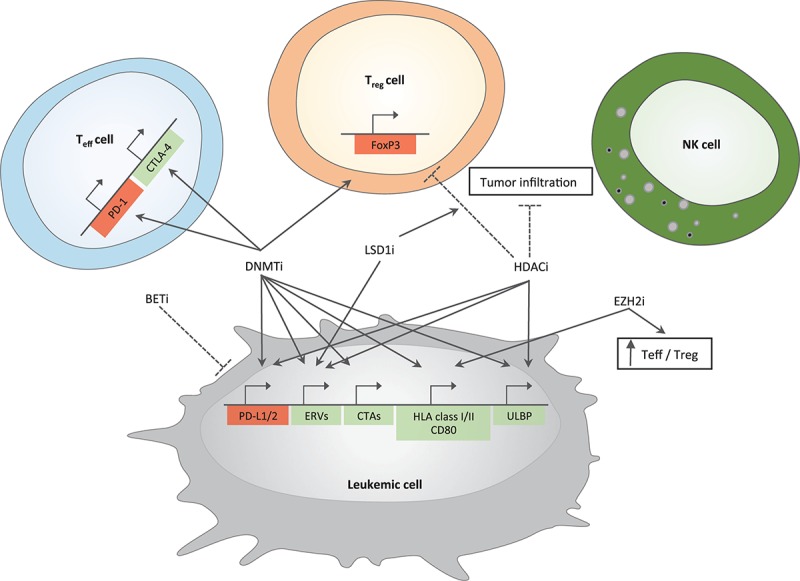
Immune-related effects of epigenetic drugs in AML. Positive and negative effects of epigenetic drugs on different immune pathways are depicted. Genes important for antitumor immunity are represented in green; in red are genes leading to inhibition of cytotoxic T cell functions. Arrows indicate induction while dotted lines represent inhibition. DNMTi and HDACi upregulate genes belonging to immune-checkpoint family (PD-L1/2) in tumor cells; DNMTi induce PD-1 and CTLA-4 in T effector cells. BETi downregulate the expression of PD-L1 on tumor cells. DNMTi together with LSD1i increase the expression of ERVs. Epigenetic drugs able to increase tumor immunogenicity include (i) DNMTi that act on cancer testis antigens (CTAs) and genes belonging to antigen presentation machinery and co-stimulation (HLA class I/II and CD80) and (ii) EZH2i, which are able to increase the expression of the latter gene family. DNMTi and HDACi can also act on ULBP gene expression, a ligand for NKG2D, an activatory receptor able to enhance NK cell functions. DNMTi upregulate FoxP3 gene expression in T regulatory cells (T_reg_ cells) while HDACi downregulate it. HDACi also reduce tumor infiltration, which is instead increased by LSD1i. EZH2i enhance antitumor immunity by increasing the number of T effector cells (T_eff_ cells) at the expense of T_reg_ cells. Mechanisms are experimentally proven in acute myeloid leukemia or inferred by studies in solid tumors.

To date, most of the studies on epigenetic drugs focused on understanding the direct effects of epigenetic therapy on tumor cells. However, interest is now extending to decipher whether epigenome rewiring may as well contribute to cancer cell eradication by triggering changes in the tumor microenvironment and in particular in the interplay between cancer cells and the immune system. In fact, the accumulation of epigenetic alterations during tumorigenesis contributes to profound changes in a plethora of transcriptional signatures including genes regulating antitumor immunity (Figure 1). As described earlier, there has been a substantial body of research showing that hypomethylating agents (such as DNMTi) act on tumor cells by counteracting hypermethylation in tumor suppressors, differentiation genes, and pathways involved in cell cycle progression. More recently, studies have highlighted a series of both positive and negative effects of epigenetic drugs on immune cells. An indirect way to unleash the immune system against AML cells is to upregulate the expression of developmental antigens in order to increase tumor antigenicity. These proteins are often regulated at the level of promoter methylation and are expressed in cancer cells due to epigenetic changes. In AML cell lines, the use of azacytidine and decitabine resulted in increased expression of the tumor-associated antigens NY-ESO-1 and WT1 and of the MAGE cancer testis antigen, with consequent activation of cytotoxic T cells (Almstedt et al., 2010; Goodyear et al., 2010). In some tumor types, including AML, the treatment with epigenetic drugs, including high doses of DNMT and HDACi, induces the reactivation of human endogenous retroviral transcripts (ERVs) (Conti et al., 2016; Daskalakis et al., 2018). ERVs are epigenetically repressed in normal somatic cells and their reactivation has been originally associated to increased genomic instability or aberrant expression of oncogenes in several cancers (Bannert et al., 2018). However, recent pieces of evidence support a role for ERV reactivation in fighting cancer in response to epigenetic therapies. Indeed, cancer cell treatment with azacytidine and decitabine leads to the cytosolic accumulation of ERV nucleic acids that in turn (i) trigger the interferon-induced viral defense pathways, (ii) boost antitumor innate and adaptive immune responses, and (iii) potentiate the effects of immune checkpoint therapies (Chiappinelli et al., 2015; Roulois et al., 2015). Similarly, gene expression analyses of transposable elements in a panel of cancer cell lines treated or not with epigenetic drugs revealed increased levels of ERVs also in response to the HDACi SB939, with an even stronger ERV reactivation observed when treating cancer cells with a combination of SB939 and decitabine (Daskalakis et al., 2018). In agreement with this observation, HDACi have been shown to induce cryptic transcription start sites encoded in long terminal repeats (Brocks et al., 2017). Whether or not HDACi exert their anticancer activity also via immune-mediated recognition of ERV nucleic acids remains to be further investigated. Finally, chemical inhibition of LSD1 was shown to concomitantly increase ERV transcription and their accumulation through reduced stability of the RNA-induced silencing complex (RISC). As a consequence, LSD1 inhibition triggers viral mimicry interferon responses, increases the infiltration of effector T cells in the tumor microenvironment, and, in preclinical models of melanoma, synergizes with the anticancer activities of immune checkpoint blockade (Sheng et al., 2018). Despite the abovementioned promising antitumor effects, further studies need to elucidate the potential impact of epigenetic therapy-mediated transposable element reactivation on cancer cell genomic instability and tumor aggressiveness.

In addition to CTAs and ERVs, genes involved in antigen presentation, both via HLA class I and class II, have been shown to be under the control of DNA methylation (Coral et al., 1999, 2013; Campoli and Ferrone, 2008). Moreover, the expression of CD80, a key co-stimulatory molecule normally absent in cancer cells, can be increased by hypomethylating agents resulting in enhanced antitumor immunity (Goodyear et al., 2010). In addition, hypomethylating agents can act also by enhancing the susceptibility of AML cells to NK cell action. In fact, azacytidine in combination with other differentiation-promoting drugs resulted in enhanced NK cell-mediated antitumor activity through the up-regulation on tumor cells of ligands for the NK cell activating receptor NKG2D (Rohner et al., 2007). Besides these positive effects on immune system activation against cancer cells, treatment of AML and MDS patients with azacytidine also led to elevated expression of a series of immune-checkpoint molecules on T cells including CTLA-4, PD-1 and its ligands (PD-L1 and PD-L2) (Yang et al., 2014). However, other studies showed that DNMT inhibitors may avoid the onset of exhaustion of cytotoxic T lymphocytes and reprogram exhausted cells into effector cells (Ghoneim et al., 2017).

Whereas it might be difficult to leverage on these immune-related effects in patients that are treatment-naive or exposed to a tight schedule of cycles of intensive chemotherapy, boosting an anti-leukemic immune response might be highly desirable in the maintenance setting, especially after allo-HCT. It is in fact well established that most of the therapeutic effect of allo-HCT relies on the transfer from the donor to the patient of a healthy immune system, capable of recognizing and eliminating residual cancer cells. Boosting this “graft-versus-leukemia” effect, possibly without unleashing severe graft-versus-host disease, has indeed over the last decades represented the “holy grail” of transplanters. For instance, the ability of DNMT inhibitors to concomitantly prime T cell-mediated anti-leukemic responses and induce T regulatory cells (Tregs) has attracted considerable interest in the allo-HCT setting (Kim and Leonard, 2007; Polansky et al., 2008; Lal et al., 2009; Feng et al., 2014). Supporting this hypothesis, in murine models of allo-HCT, the use of hypomethylating agents suppresses T cell proliferation and cytokine production, resulting in reduced graft versus host disease without any effect on graft versus leukemia (Choi et al., 2010; Sanchez-Abarca et al., 2010). Similar results were also reported in AML patients, where post-transplantation treatment with azacytidine was associated with an increase in the number of Tregs (Goodyear et al., 2012). Indeed, trials testing azacytidine in the prophylactic (de Lima et al., 2010; Craddock et al., 2016a) and pre-emptive (Platzbecker et al., 2012) settings have reported encouraging results in terms of both overall survival and donor chimerism stabilization.

Interestingly, azacytidine, either alone or in combination with donor lymphocyte infusions (DLIs), has shown interesting results also in the more challenging setting of the treatment of AML patients with frank hematological relapse. To date, data on more that 600 patients have been collected, reporting very variable results in terms of clinical outcomes (Lubbert et al., 2010; Schroeder et al., 2013, 2015; Steinmann et al., 2015; Ghobadi et al., 2016; Craddock et al., 2016b). Starting from these heterogeneous results and lack of consent on treatment schedules, two retrospective surveys have been performed in more homogeneous cohorts of patients, with the aim of identifying prognostic factors for treatment response and long-term survival. These studies reported that patients with the highest benefit from azacytidine + DLI combination were those transplanted in complete remission, who presented with low disease burden at the time of relapse (molecular relapse or blasts <20% in bone marrow) and that experienced a longer remission time from HCT to overt relapse (Schroeder et al., 2015; Craddock et al., 2016a). Even if not so well substantiated, studies reporting the efficacy of decitabine after allo-HCT showed similar results (Han et al., 2015; Pusic et al., 2015; Schroeder et al., 2018).

Like DNMT inhibitors, small molecules targeting HDAC activity are able to induce positive and negative immunomodulatory effects; in fact, they can increase cancer immunogenicity through the expression of HLA class I genes and tumor antigens and/or block T cell response by the upregulation of ligand for checkpoint inhibitor receptors as well as by reducing the number of Tregs (Setiadi et al., 2007; Shen et al., 2012; Woods et al., 2015).

As a result of the evidence that both DNMT and HDACi can contribute to T cell exhaustion, a number of clinical studies are currently testing the combination of these epigenetic drugs with immune checkpoint inhibitors (Daver et al., 2019).

With these immune-related effects in mind, HDACi alone or in combination with azacytidine and DLI have been tested in a post-transplantation setting. Results on phase I/II trials testing the combinatorial treatment as maintenance therapy after transplantation reported a 2-year rate overall survival and a relapse free survival rate of 81 and 75%, respectively, (Bug et al., 2017).

Besides their reported effect in suppressing tumor cell proliferation, recently published preclinical studies highlighted the role of EZH2 inhibitors in enhancing antitumor immunity by altering the ratio between effector and regulatory T cells in favor of the first population (Wang et al., 2018). Another interesting property of these drugs is the ability to restore HLA class I and II expression in EZH2-mutated cases of DLBCL (Ennishi et al., 2019), rendering them promising candidates to be tested in the post-transplantation setting.

## Discussion

AML is a highly heterogeneous cancer type often associated with bad prognosis, with the minority of patients being cured without allo-HCT and for which new therapeutic approaches are urgently needed. Here, we provide a comprehensive overview of studies documenting the effects of epigenetic aberrations in AML pathogenesis and summarized the most recent clinical efforts reporting safety and efficacy of epigenetic compounds for AML treatment. By targeting multiple pathways simultaneously, epigenetic drugs hold great therapeutic promise, although several challenges need to be faced to see in the clinics the full success of such a therapy. One of the most clinically relevant issue still limiting the full implementation of epigenetic therapies in the treatment algorithms for AML patient relates to our incomplete understanding of their interactions with chemo- and immunotherapies. Understanding synergistic and antagonistic effects of epigenetic drugs with current therapeutic paradigms will be fundamental to design new trials incorporating epigenetic therapies based on a specific biological rationale, possibly associated to ancillary studies aimed at determining their effects on leukemic cells and the immune system. For this purpose, it will be necessary to develop new models to test epigenetic therapies, possibly employing actual primary patient samples instead of cell lines and mouse models, that poorly recapitulate the complexity of such a heterogeneous disease. Very recently, the group of Melnick developed a platform to systematically study long-term effects of epigenetic drugs as monotherapy and in combination on a large number of *ex vivo* cultured primary AML (Duy et al., 2019). Other suitable models known to better mirror AML complexity are next-generation patient-derived xenograft (PDX) models and humanized niche xenograft models (Antonelli et al., 2016; Reinisch et al., 2016; Abarrategi et al., 2017). Although the stability of the epigenetic landscape of primary AML samples in these models remains to be experimentally tested, they can provide a valid alternative to monitor the long-term biological consequences of epigenetic treatments and study their impact on tumor cells as well as on several components of the tumor microenvironment. Given the abovementioned impact of epigenetic therapies in modulating antitumor immunity, the same preclinical *ex vivo* and *in vivo* models will be instrumental to also test epigenetic drugs in combinations with immunotherapy to further enhance their antitumor efficacy. Of note, most epigenetic regulators will have an impact on transcriptional programs fundamental also for normal cell fitness, posing the risk for increased toxicity. Accurate selection of patients that will benefit from a given epigenetic therapy is thus needed to guide a proper choice of epigenetic therapies in AML patients. Additionally, the concomitant presence of distinct leukemia subclones may further challenge the success of epigenetic therapies for AML treatment. The advent of NGS technologies mapping all the different layers of epigenetic regulation at a single-cell level together with transcriptomic and genomic analysis will help to better address these questions in the setting of AML. Altogether, we believe that the application of innovative technologies and more suitable preclinical models that take into consideration the interplay between cancer cells and the immune system will, in the near future, better elucidate biological processes on the basis of tumor response to epigenetic therapy and contribute to move one step forward toward personalized medicine for cancer treatment.

## Author Contributions

All authors contributed to writing the review and discussed its contents.

## Conflict of Interest

The authors declare that the research was conducted in the absence of any commercial or financial relationships that could be construed as a potential conflict of interest. The reviewer IG declared a past co-authorship with one of the authors LV to the handling Editor.
